# The gastrointestinal-brain-microbiota axis: a promising therapeutic target for ischemic stroke

**DOI:** 10.3389/fimmu.2023.1141387

**Published:** 2023-06-02

**Authors:** Yan-hao Wei, Ren-tang Bi, Yan-mei Qiu, Chun-lin Zhang, Jian-zhuang Li, Ya-nan Li, Bo Hu

**Affiliations:** Department of Neurology, Union Hospital, Tongji Medical College, Huazhong University of Science and Technology, Wuhan, China

**Keywords:** ischemic stroke, brain, inflammation, gastrointestinal tract, gastrointestinal microbiota

## Abstract

Ischemic stroke is a highly complex systemic disease characterized by intricate interactions between the brain and gastrointestinal tract. While our current understanding of these interactions primarily stems from experimental models, their relevance to human stroke outcomes is of considerable interest. After stroke, bidirectional communication between the brain and gastrointestinal tract initiates changes in the gastrointestinal microenvironment. These changes involve the activation of gastrointestinal immunity, disruption of the gastrointestinal barrier, and alterations in gastrointestinal microbiota. Importantly, experimental evidence suggests that these alterations facilitate the migration of gastrointestinal immune cells and cytokines across the damaged blood-brain barrier, ultimately infiltrating the ischemic brain. Although the characterization of these phenomena in humans is still limited, recognizing the significance of the brain-gastrointestinal crosstalk after stroke offers potential avenues for therapeutic intervention. By targeting the mutually reinforcing processes between the brain and gastrointestinal tract, it may be possible to improve the prognosis of ischemic stroke. Further investigation is warranted to elucidate the clinical relevance and translational potential of these findings.

## 1 Introduction

Stroke represents a significant global burden in terms of mortality and disability. It is currently ranked as the second leading cause of death and the third leading cause of disability worldwide (1). Stroke can be categorized into two major types: ischemic stroke (IS) and hemorrhagic stroke. IS occurs when cerebrovascular diseases disrupt blood supply to the brain, leading to ischemic hypoxic necrosis in brain tissues. Consequently, a cascade of inflammatory reactions occurs, causing damage to the brain and the blood-brain barrier (BBB) during the early stages of IS. This process establishes major channels of communication between the brain and the gastrointestinal tract (GT), initiating frequent interactions and contributing to a poor prognosis of stroke.

In clinical practice, current treatments for ischemic stroke primarily involve thrombolysis and embolectomy. However, each treatment modality comes with its own specific requirements. Intravenous thrombolysis necessitates the administration of recombinant tissue plasminogen activator (rtPA) within a strict time window of 4.5 hours from the onset of stroke symptoms. On the other hand, mechanical thrombectomy is indicated for patients with large artery occlusion who can undergo the procedure within 6 hours of symptom onset (2). However, some patients experience poor treatment outcomes and prognoses (3, 4). Therefore, there is an urgent need to explore novel therapeutic strategies.

In recent years, there has been a growing interest in understanding the bidirectional communication and interaction between the brain and GT following ischemic stroke, leading to the emergence of the concept known as the gut-brain-microbiota axis (GBMA). Multiple animal experiments and clinical evidence now suggest that there is mutual communication and interaction between the post-stroke brain and GT (5–10).

Stroke can lead to complications such as impaired intestinal barrier function, microbiota translocation, and post-stroke infections, while cellular debris and brain-derived antigens activate the intestinal immune system (5, 11). The activated immune cells and cytokines then infiltrate the ischemic brain, exacerbating stroke injury and perpetuating a vicious cycle that culminates in a significant deterioration of stroke prognosis (5, 11).

Therefore, closely monitoring the post-stroke crosstalk between the brain and GT is crucial for identifying potential therapeutic strategies that can terminate this vicious cycle, ultimately leading to improved stroke prognosis. This review aims to provide a comprehensive overview of the crosstalk between the brain and GT following ischemic stroke, elucidate the underlying mechanisms by which the brain and GT interact to worsen stroke outcomes, and discuss potential therapeutic strategies that may enhance stroke prognosis.

## 2 Stroke onset and crosstalk between the brain and gastrointestinal tract

After a stroke, brain tissues experience ischemia and hypoxia, leading to cellular edema and neuronal dysfunction. If blood supply is not promptly restored, brain cells will undergo irreversible damage, resulting in the death of brain tissues. Even when blood supply is quickly restored, an increase in reactive oxygen species (ROS) and free radicals during reperfusion can over-activate the inflammatory response, causing secondary brain injury and further activation of local immune cells, which leads to inflammation in the brain tissues (12). The occurrence and progression of local inflammation in the ischemic brain then contribute to the expansion of the infarct and the recruitment of central microglia and peripheral immune cells, resulting in a more robust inflammatory response (13–15).

The GBMA, proposed in recent years, refers to a bidirectional regulatory axis comprising the brain, GT, and gastrointestinal microbiota (GM). Numerous studies have demonstrated the significant impact of GBMA on the pathophysiological processes of IS (16). For example, research suggests that stroke can rapidly induce dysbiosis in the gastrointestinal microbiota, which, in turn, exacerbates brain infarction. This bidirectional relationship between gut dysbiosis and stroke highlights the intricate interplay between the gut and the brain (11).

The script of GBMA after stroke encompasses three main subjects:

The brain as the main subject: On the one hand, ischemic brain tissues after stroke produce local neuroinflammation (12). The activation of matrix metalloproteinases (MMPs) and the increase in ROS lead to the disruption of the BBB, facilitating the outward migration of damage-associated molecular patterns (DAMPs), injured brain-secreted antigens, exosomes, and the inward migration of peripheral immune cells (17). On the other hand, the brain receives various signals after stroke onset, which affect the homeostasis of the gastrointestinal microenvironment. These effects include increased gastrointestinal permeability and alterations in the composition and translocation of gastrointestinal microbiota, mediated by neurotransmission (18), neuroendocrine (19), and other mechanisms.

The gastrointestinal immune system as the main subject: On the one hand, the GT serves as a vital immune organ, housing a significant number of immune cells that account for more than 70% of the entire immune system. Therefore, it acts as a crucial source of recruited immune cells in the ischemic brain tissue (20).

Immune cells in the GT, activated by DAMPs, injured brain-secreted antigens, and exosomes, release a plethora of cytokines. Subsequently, these immune cells and cytokines migrate through the damaged BBB and infiltrate the ischemic brain tissues, facilitated by chemokines, exacerbating neuroinflammation (21). On the other hand, the activation of gastrointestinal immunity can also affect the homeostasis of the gastrointestinal microenvironment (22).

The gut microbiota as the main subject: On the one hand, the translocation of gastrointestinal microbiota intensifies the degree of systemic inflammation, thereby significantly worsening patient prognosis (23, 24). Furthermore, the composition of gastrointestinal microbiota is closely linked to risk factors for stroke (5). On the other hand, the presence of gastrointestinal microbiota has specific effects on gastrointestinal immune cells (25).

**Figure 1:**
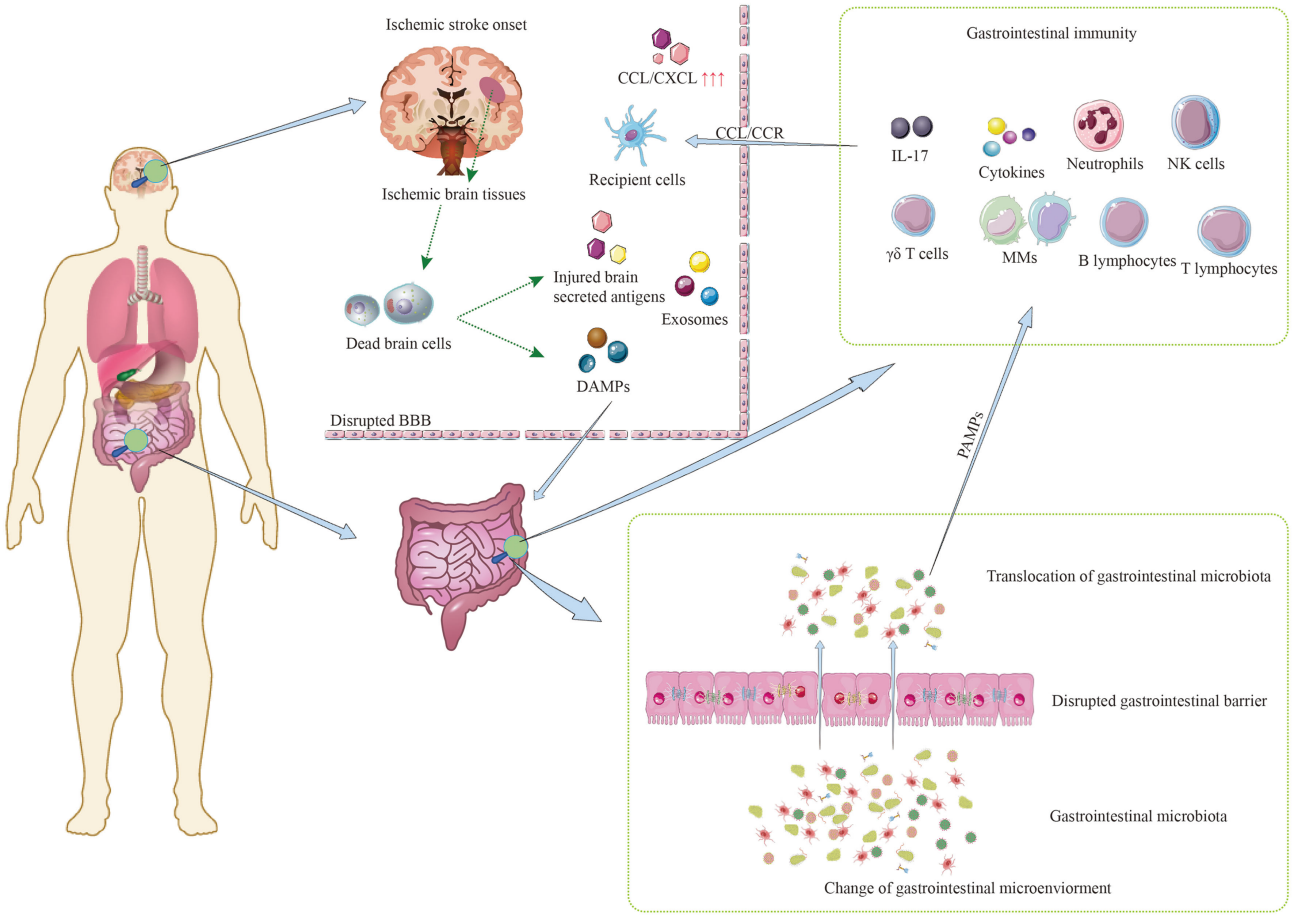
Mutual communication among three main subjects after ischemic stroke.

In conclusion, the interaction among these three subjects initiates after ischemic stroke, involving a complex interplay of various pathophysiological events (**Figure 1**). While certain processes can contribute to a detrimental cycle, such as the activation of inflammatory cascades, it’s also important to note that protective immune subpopulations play a role in promoting tissue recovery after the injury. Therefore, the overall impact of these interactions on stroke outcomes is multifaceted and involves both detrimental and protective elements. Understanding and modulating these interconnected processes may hold the key to improving the prognosis associated with ischemic stroke.

## 3 Compositional interactions of GBMA after ischemic stroke

**Figure 2:**
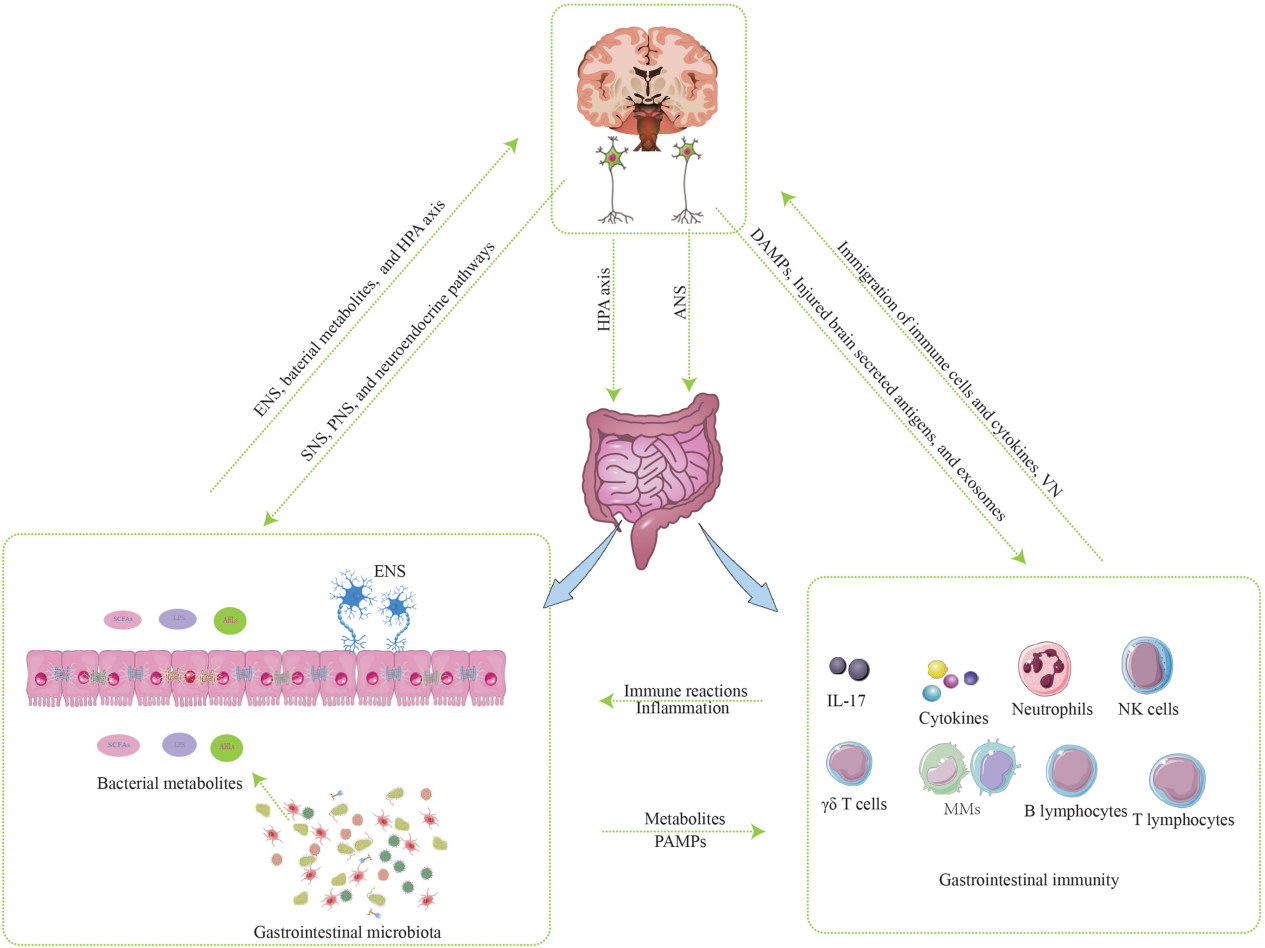
The mechanisms of interaction between the brain and gastrointestinal tract.

GBMA is a bidirectional communication system composed of the brain, GT, and GM. Three components of the bidirectional communication system interact and participate in the occurrence, development, and prognosis of IS. The mechanisms of interaction between the brain and GT are as follows (**Figure 2**).

### 3.1 Components interact via neurotransmission

The neurotransmission within the GBMA is primarily mediated by the enteric nervous system (ENS), the autonomic nervous system (ANS), and the vagus nerve (VN). The central nervous system (CNS) integrates information from the brain and spinal cord and transmits signals to the ENS and various gastrointestinal cells, including smooth muscle cells, blood vessels, and gland cells, through the ANS or neuroendocrine pathways. These inputs of signals modulate gastrointestinal motility, permeability, innate immune cell activation, secretion of the GT, and composition of the gastrointestinal microbiota (26). For example, brain injury after stroke causes sympathetic stimulation of the cecum to release norepinephrine, which is accompanied by a decrease in mucin secretion (27). Changes in sympathetic nerve activity can also lead to changes in cecal bacterial communities, and the degree of changes is correlated with the severity of brain injury (27). In the post-stroke mice, the total neuron and nerve fiber numbers were not unchanged compared with the sham group, but the ratio of adrenergic neurons to cholinergic neurons was elevated after IS, suggesting a misbalance between adrenergic and cholinergic signaling within the submucosal plexus in the GT following IS (18). In addition, the elevated levels of catecholamines in the GT after IS are associated with damage of the gastrointestinal mucosal barrier (18). These findings suggest that post-stroke alterations in adrenergic and cholinergic signals received by the ENS, resulting in the damage of gastrointestinal microenvironment.

Moreover, the ENS interacts with both the GT endocrine and immune systems and plays a critical role in nutrient absorption and guaranteeing the integrity of the mucosal barrier (18). Additionally, the ENS includes the myenteric plexus and submucosal plexus, which integrate and process various gastrointestinal signals and directly transmit the information to the CNS via the VN (28). Furthermore, some studies show that the VN is also the primary way for the gastrointestinal microbiota to affect the function and metabolism of CNS.

### 3.2 Components interact via neuroendocrine

The activation of the hypothalamic-pituitary-adrenal (HPA) axis, a critical neuroendocrine system, occurs in response to vagal afferent fibers during stroke, triggering the release of cortisol from the adrenal cortex (19). Currently, there is insufficient evidence to substantiate the hypothesis that elevated cortisol levels directly cause an ecological imbalance in the gastrointestinal tract in the context of stroke. However, stress can enhance intestinal permeability through mast cell activation mediated by corticotropin-releasing hormone (CRH) (29). Additionally, the HPA axis, via stimulation of β-adrenergic receptors of sympathetic nervous system (SNS), increases gastrointestinal permeability, promotes the generation of Th17 cells in the GT and bring about alterations in the gastrointestinal microbiota composition in the context of stressful stimuli (30, 31). These changes in gastrointestinal permeability and microbiota composition trigger the release of inflammatory mediators, including pro-inflammatory cytokines, microbial antigens, and prostaglandins, capable of crossing the BBB and activating the HPA axis (32). These findings suggest that the HPA axis offers bidirectional communication in the GBMA. However, further research is needed to validate the association between HPA axis activation and gastrointestinal ecological imbalance following stroke. Moreover, it remains to be elucidated whether stroke acts as a stressor that initiates a stress response, subsequently leading to HPA axis activation, or if stroke directly triggers HPA axis activation.

Enterochromaffin cells (ECs), specialized cells found in the gastrointestinal tract, play a crucial role in the secretion of 5-hydroxytryptamine (5-HT), a multifunctional molecule (33). Acting upon G protein-coupled receptors (GPCRs) of gastrointestinal cells, 5-HT influences various aspects such as gastrointestinal secretion, motility, permeability, and inflammation (33). Although 5-HT cannot directly penetrate the BBB to exert an impact on the brain, it modulates the VN and contributes to gastrointestinal inflammation (34). Additionally, a few specialized bacterial and gastrointestinal metabolites, such as γ-aminobutyric acid, deoxycholic acid, and tyramine, have been found to induce the synthesis and secretion of 5-HT by stimulating GPCR5 of ECs (35).

### 3.3 Components interact via immune reactions

Ischemic brain tissues release DAMPs, injured brain-secreted antigens, and exosomes, which activate microglia, leading to increased production of pro-inflammatory cytokines and chemokines, as well as recruitment and infiltration of immune cells into the ischemic brain (36). These DAMPs, antigens, exosomes, and pro-inflammatory cytokines cross the disrupted BBB and enter the peripheral circulation. Then elevated levels of DAMPs and pro-inflammatory cytokines in circulation contribute to increased gastrointestinal permeability, leading to the translocation of gastrointestinal microbiota and the development of gastrointestinal dysbiosis (26, 37). Furthermore, DAMPs, injured brain-secreted antigens (38, 39), and exosomes (40, 41) activate the immune cells in the GT, triggering peripheral immune reactions. Moreover, gastrointestinal innate immune cells, such as neutrophils, monocyte macrophages (MMs), natural killer (NK) cells, and γ-δ T cells, respond rapidly and utilize the CCR-CCL pathways to cross the BBB and infiltrate ischemic brain tissues within hours (42, 43).Then these immune cells cause local and systemic inflammatory and immune reactions (37, 44). The activated immune cells produce inflammatory mediators that recruit additional immune cells, further exacerbating neuroinflammation (21). The progression of neuroinflammation leads to worsened brain tissue damage and increased release of DAMPs, injured brain-secreted antigens, and exosomes into the peripheral circulation, thereby exacerbating the deterioration of the gastrointestinal microenvironment.

Additionally, the translocation of gastrointestinal microbiota into the circulation generates pathogen−associated molecular patterns (PAMPs), which activate pattern−recognition receptors (PRRs) and trigger bacterial inflammation and immune reactions (45, 46). In an animal experiment, all mice with middle cerebral artery occlusion (MCAO) developed spontaneous bacterial infection after three days, with > 95% of the cultured bacteria identified as E. coli, while no signs of pneumonia were observed in the sham-operated group, ruling out surgical stress as a trigger (47). Another study detected lipopolysaccharide (LPS) in ischemic brain tissue following stroke (48). These findings suggest that PAMPs begin to mediate bidirectional communication of GBMA during the non-acute phase after stroke. Therefore, it can be concluded that prior to gastrointestinal dysbiosis and GM translocation, inflammatory and immune reactions are solely mediated by DAMPs. Initial inflammatory activation caused by DAMPs is acute and first to occur during brain damage. However, following GM translocation, both DAMPs and PAMPs contribute to the inflammatory and immune reactions (45, 46). Furthermore, as a protective mechanism for the brain, systemic immunosuppression (SIS) occurs quickly and involves the SNS, VN, and HPA axis. However, the protective mechanism also facilitates the exacerbation of inflammatory responses after GM translocation.

### 3.4 Components interact via gastrointestinal bacterial metabolites

Gastrointestinal bacterial metabolites influence GBMA by binding to gastrointestinal receptors and parenteral receptors as well as intervening in the three pathways mentioned above.

Short-chain fatty acids (SCFAs) are a class of crucial gastrointestinal bacterial metabolites that significantly impact stroke pathophysiology by modulating systemic and intracerebral immune cells. Multiple studies have confirmed that SCFAs readily cross the BBB and exerts effects on post-stroke recovery by influencing immune cells within the brain and the entire body (49–51). SCFAs may exert their effects through the following mechanisms: (1) SCFAs, a kind of inhibitor of histone deacetylases, act on the immune cells by blocking histone deacetylation to influence gene expression (52, 53). For example, HDAC inhibition with SCFAs downregulated IL-17 expression and increased the production of Foxp3^+^, CD4^+^, and CD25^+^, which induced the proliferation of CD4^+^ and Tregs and promoted neuroprotection after IS (54–56). (2) SCFAs work as signaling molecules binding to the GPCR of immune cells’ surface to activate immune cells. Binding of SCFAs to the GPR41/43 of ECs and phagocytes in the GT leads to the rapid production of cytokines and chemokines, mediating systemic inflammation (57). Additionally, SCFAs decrease IL-6 and IL-8 production by activating endothelial cells through acting on the GPR41/43, exerting anti-inflammatory effects of activated (58). (3) SCFAs contribute to strengthening the gastrointestinal mucosal barrier by regulating mucus secretion from gastrointestinal epithelial cells and repairing injured intestinal mucosa through increased expression of tight junction proteins, thereby decreasing the GM translocation (59, 60). (4) SCFAs activate the SNS by binding to GPR41 of sympathetic neurons, crossing the BBB and affecting the neural signaling and neurotransmitter production (49, 61). (5) SCFAs promote the expression of the tryptophan hydroxylase 1 (TPH1) gene in ECs, leading to increased secretion of 5-HT, which impacts gastrointestinal motility, permeability, and inflammation (33, 62). These findings highlight the critical role of SCFAs in the immunomodulation, post-stroke pathophysiology, and recovery.

LPS, a primary component of the gram-negative bacterial outer membranes, is an inflammatory stimulus that triggers immune responses through toll-like receptors (TLR) activation, thereby contributing to gastrointestinal dysbiosis (48). There is study have shown the presence of LPS in ischemic brain tissues, neurons, and microglia following stroke, with the higher levels of TLR4 (LPS receptor) and inflammatory cytokines in the ischemic brain tissues compared to control groups (63). Therefore, LPS may enter the blood circulation and ischemic brain tissues by crossing the disrupted gastrointestinal barrier and BBB after stroke. Furthermore, LPS induced endotoxemia exacerbates post-stroke neuroinflammation by increasing pro-inflammatory cytokines levels in the blood circulation (63). Animal experiments have indicated that TLR4 knockout may confer neuroprotective effects in mice with ischemic brain injury (64). Additionally, LPS directly activates the HPA axis by binding to TLR4 on human adrenal cells, stimulating cortisol secretion (65). Moreover, Clinical studies have shown elevated LPS levels in circulation after ischemic stroke, with high levels associated with poor short-term prognosis in stroke patients (66). Another clinical study suggests that dysbiosis of gut microbiota and elevated LPS levels increase the risk of poor functional outcomes in acute ischemic stroke patients, highlighting their potential as prognostic markers and therapeutic targets (67). These findings underscore the importance of LPS as a mediator in the interaction between GT and brain after stroke. Therefore, regulating the level of LPS may be a useful therapy for treating IS.

Aryl hydrocarbon ligands (AHLs) are another active metabolite derived from tryptophan. Intestinal epithelial cells (IECs) express the aryl hydrocarbon receptor (AHR), and AHLs activate the AHR of IECs, contributing to the renewal of gastrointestinal barrier epithelial cells, thus ensuring their integrity and normal function (68). For example, the AHR agonist FICZ reduces the damage to gastrointestinal mucosal barrier function (69). Moreover, AHLs maintains the expression and distribution of tight junction protein ZO-1 to improve the increase of intestinal permeability induced by hypoxia (69). AHLs also regulate the immune functions of gastrointestinal immune cells by acting on AHR. Microbiota-derived indoles, a type of tryptophan metabolite, act on the AHR of immune cells in the GT, inducing the production of IL-22, which is beneficial for the maintenance of gastrointestinal homeostasis (70). AHR also modulates neutrophil function and promotes the secretion of IL-17 and IL-22 (71). Tryptophan metabolites drive neuroprotection by activating the AHR of astrocytes (72). Gastrointestinal macrophages are also involved in AHR mediated maintenance of gastrointestinal epithelial integrity (73). In addition to acting locally in the GT, AHLs can also cross BBB, exerting effects on the CNS. For example, AHR expression in microglia is upregulated after IS and contributes to the development of neuroinflammation and subsequent brain parenchymal edema (74). And the use of the AHR antagonist CH223191 reduces the severity of neurological impairment (74). Although AHLs and AHR have been extensively studied in the context of gastrointestinal diseases, their role in stroke is relatively understudied, and further mechanistic research is needed.

Vitamin B12 (VB12) can be obtained from animal derived foods or can be provided by certain gastrointestinal microbiota (75). Some studies have demonstrated that exogenous VB12 supplementation stimulates the production of SCFAs by GM, indirectly involved in the effect of SCFAs on the GBMA (76). VB12 is essential for the synthesis of methionine and nucleotides. VB12 deficiency leads to the accumulation of homocysteine, promoting atherogenesis and the risk of ischemic stroke. Homocysteine accumulation alters immune homeostasis, promoting inflammation, inducing MCP-1 and IL-8 secretion, and enhances endotoxin-induced activation monocytes by activating nuclear factor κ B (77, 78). Additionally, VB12 deficiency hinders the recovery of neurological function after ischemic stroke due to the decrease nucleotide synthesis (79).

Besides the aforementioned microbial metabolites, various other gastrointestinal microbiota-derived metabolites, such as γ-aminobutyric acid, norepinephrine, tyramine, and dopamine, participate in the communication between the brain and GT by acting directly on brain cells or indirectly through interactions with vagal afferent fibers. In conclusion, gastrointestinal bacterial metabolites play a crucial regulatory role in the post-stroke immune system. A deeper understanding of the gastrointestinal microbiota and their metabolites is required to develop a novel therapeutic strategy for stroke treatment.

## 4 The impact of ischemic stroke on GT

Dysbiosis occurs in the GT following ischemic stroke due to various mechanisms mentioned previously, including increased gastrointestinal permeability resulting from disruption of the gastrointestinal barrier, translocation and alteration of the gastrointestinal microbiota, post-stroke infections, and other GT complications.

### 4.1 Gastrointestinal permeability increases after stroke

The GT encompasses three key entities: the gastrointestinal barrier, gastrointestinal microbiota, and the mucosal immune system. The gastrointestinal barrier and the mucosal immune system consist of many immune cells and lymphoid tissues, which protect the normal tissues of GT from pathogenic microorganisms (80). The gastrointestinal barrier has the critical physiological function that separates normal gastrointestinal tissues from toxic luminal contents (81). Among the components influencing the permeability of the gastrointestinal barrier, intercellular junctional complexes play a significant role. These complexes tightly connect intestinal epithelial and endothelial cells and are highly dynamic regions. Various external and intracellular stimuli, bacterial toxins, cytokines, hormones, and drugs, can impact the permeability of intercellular junctional complexes (82, 83).

Numerous studies have reported that activating the SNS and HPA axis increases gastrointestinal permeability after IS (5, 27, 84). Both young and old mice subjected MCAO exhibited a significant increase in gastrointestinal vascular and epithelial permeability from 90 minutes to 24 hours post-stroke, with aged mice demonstrating more pronounced intestinal leakage (23). This could be associated with the decreased distribution of ZO-1, a tight junction protein in the GT. For example, the research carried out by Stanley et al. revealed a quantitative decrease in ZO-1 expression in the stroke group compared to the sham group, indicating damage to the gastrointestinal barrier and the increase in gastrointestinal permeability (18). However, it is unclear whether stroke will affect other intestinal tight junctions, such as occludin, vascular endothelial cadherin, β-catenin, and junctional adhesion molecule-A (85). Additionally, certain gastrointestinal bacterial metabolites as mentioned earlier, also influence in the gastrointestinal barrier. For example, bacterial-derived indole enhances epithelial tight junction integrity and reduces inflammation (86). Moreover, stroke affects the composition and metabolic activity of the gastrointestinal microbiota, potentially compromising the function of gastrointestinal epithelial cells, thereby influencing their structure and function and increasing gastrointestinal permeability and translocation of GM (87). For example, the reduction of intestinal goblet cells, crucial for intestinal homeostasis, may impair the formation of the intestinal mucus barrier and promote the translocation of intestinal microbiota (88).

In conclusion, stroke disrupts the function of the gastrointestinal barrier, allowing harmful substances to cross the gastrointestinal mucosa, enter the bloodstream, and eventually reach the brain through the damaged BBB, thereby exacerbating stroke prognosis. Therefore, protecting gastrointestinal barrier function following stroke might represent a potential target for improving stroke prognosis.

### 4.2 Stroke-induced translocation of gastrointestinal microbiota and post-stroke infection

The gastrointestinal tract harbors a complex community of over 1000 species of commensal microbiota (89). After the gastrointestinal barrier is damaged, the gastrointestinal microbiota and toxic luminal contents cross the damaged gastrointestinal barrier into the circulation, potentially serving as the source of systemic inflammation or systemic inflammatory response syndrome (90). Moreover, increasing evidence from experimental and clinical studies suggest that IS can induce alterations in the composition of gastrointestinal microbiota (**Table 1**). Various clinical studies and animal experiments have observed a decrease in the abundance of bacteria responsible for SCFAs production in the intestine to varying degrees (5, 8, 93, 94). For example, the occurrence of dysbiosis in the gastrointestinal microbiota following stroke has been well-documented. It involves a disruption in the balance of microbial populations, characterized by decreased beneficial bacteria and an overgrowth of potentially harmful bacteria (11). However, the precise molecular mechanisms underlying the changes in intestinal microbial composition have yet to be elucidated.

**Table 1:** Changes of gastrointestinal microbiota in the clinical studies and animal experiments.

Changes in gut microbiota: clinical studies.		
Changes	Findings	Reference
Counts of Lactobacillus and Atopobium are increasing Counts of the Lactobacillus sakei subgroup are decreasing	IS induce the altered composition of gut microbiota and decrease the levels of fecal acetic acid.	(91)
Abundance of pathogenic bacteria such as Enterobacteriaceae are increasing Abundance of butyrate producer such as Faecalibacterium are decreasing	Stroke Dysbiosis Index (SDI) is used to measure gut microbiota dysbiosis after IS, which is significantly correlated with patients’ outcome	(92)
Pathogenic bacteria (Enterobacteriaceae and Porphyromonadaceae), Lactobacillaceae and Akkermansia are increasing. SCFAs producing bacteria (Lachnospiraceae Bacteroides, Anaerostipes, Roseburia, Faecalibacterium and Blautia) are decreasing.	The SCFAs levels were negatively correlated with stroke severity. Reduced SCFAs levels were associated with an increased risk of poor functional outcomes.	(8)
Changes in gut microbiota: animal experiments.		
Changes	Findings	Reference
Firmicutes, Bacteroidetes, and Actinobacteria are increasing Species diversity decrease significantly	Restoring the balance and homeostasis of gut microbiota is beneficial to the treatment of IS.	(5)
Pathogens or opportunistic pathogens, such as Shuttleworthia, Bacteroides, Alistipes, Klebsiella, Haemophilus, Fusobacterium, Proteus, Papillibacter and Faecalibacterium are increasing	Remodeling the gut microbiota can regulate the brain–gut barriers and relieve the gut microbiota dysbiosis.	(6)
Levels of Prevotellaceae is decreasing Levels of Peptococcaceae is increasing	Ischemic brain injury induces profound changes in the gut microbiota.	(27)

**Figure 3:**
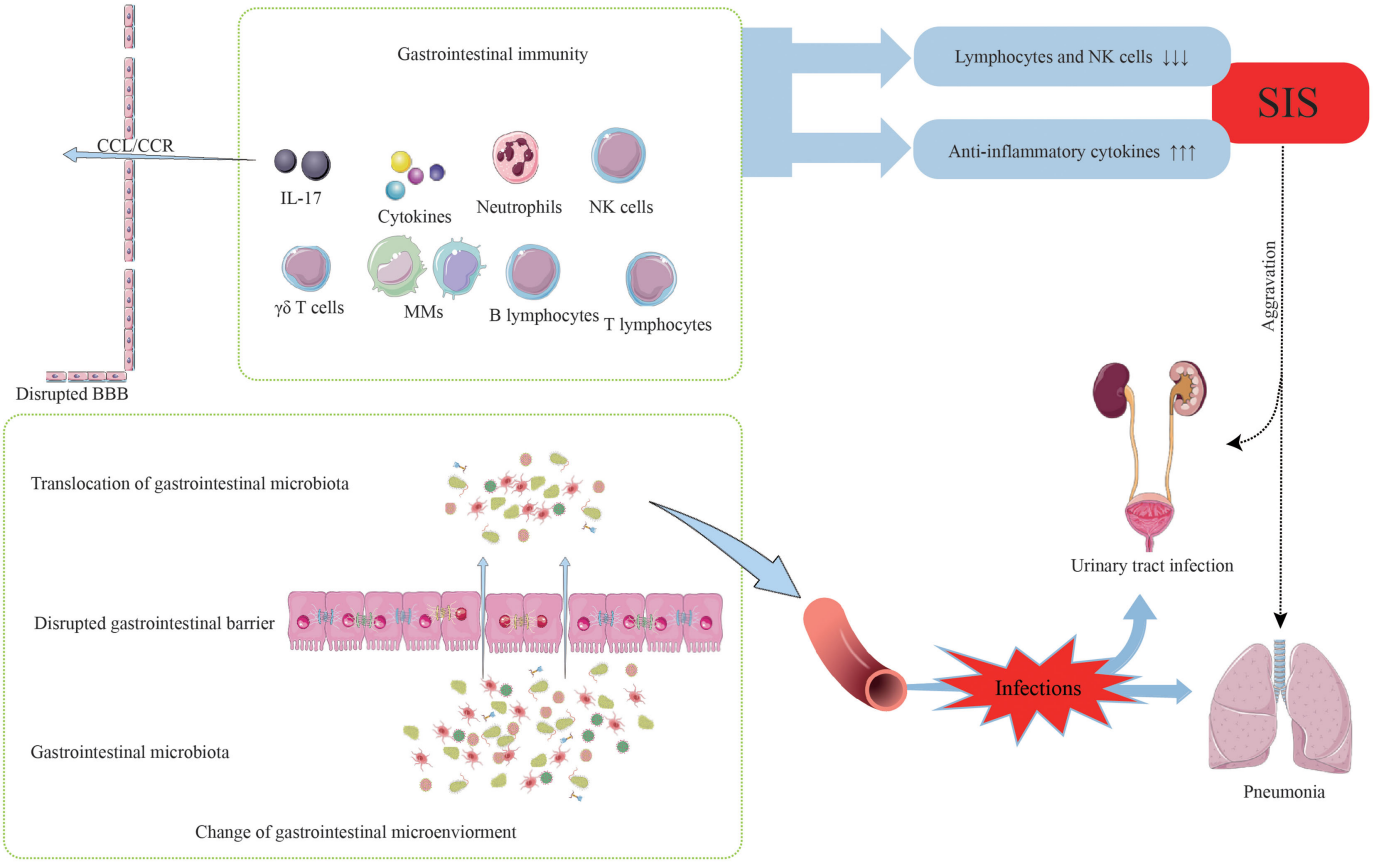
The occurrence of post-stroke infections.

Gastrointestinal bacteria translocate into the blood circulation following disruption of the gastrointestinal barrier. Subsequently, these bacteria have the potential to invade normal sterile tissues and internal organs via the bloodstream, giving rise to gut-derived sepsis and infections at various sites (24, 95). Moreover, individuals who have experienced ischemic stroke are more susceptible to post-stroke infections, which can be attributed to the systemic immunosuppressed state (96). The systemic immunosuppression (SIS) manifests approximately 24 hours after stroke onset and characterized by decreased levels of lymphocytes and NK cells, and increased levels of anti-inflammatory cytokines in the peripheral circulation (97). The underlying mechanisms of SIS are thought to involve complex pathways, including the SNS, VN, and HPA axis, through which CNS communicates with the peripheral immune system (97, 98). The occurrence of SIS at 24 hours post-stroke, accompanied by a significant reduction in circulating lymphocyte count, may contribute to an increased risk of infections (99). Indeed, post-stroke infectious complications are frequently observed in stroke cases, with pneumonia and urinary tract infections being the most common, and pneumonia has been shown to significantly contribute to mortality after stroke (18, 100). Notably, many of the detected common infecting microbes following stroke are gastrointestinal commensal bacteria, indicating that the translocation of gastrointestinal microbiota after stroke may play a role in post-stroke infections (**Figure 3**) (18). In addition to the abovementioned complications, many other gastrointestinal complications may occur, such as dysphagia, incontinence, constipation, gastrointestinal bleeding, and deep vein thrombosis (101–104).

In summary, stroke disrupts the original balance between the CNS and the gastrointestinal immune system, thereby resulting in the occurrence and development of post-stroke infection. The mechanisms underlying post-stroke infections require further investigation. Protecting the gastrointestinal barrier function from damage, promoting its recovery following stroke, and impeding the migration of harmful microbiota across the damaged barrier are important directions for future efforts.

## 5 The impact of GT on ischemic stroke

After stroke, there is evidence suggesting the disruption of the gastrointestinal barrier, translocation of gastrointestinal microbiota (GM), and activation of the gastrointestinal immune system, which may simultaneously impact the pathophysiological development of ischemic stroke and related risk factors. It is important to note that the supporting evidence primarily stems from experimental studies, and while some studies have provided corroborative results, there may be variability among the findings. Therefore, a critical examination of the existing evidence and its implications is warranted. Next, the effect of the GT on IS will be discussed from three aspects.

### 5.1 DAMPs induced aseptic inflammation and ischemic stroke

Ischemic brain tissues release DAMPs, often resulting in altered gastrointestinal immune homeostasis. Altered gastrointestinal immune homeostasis regulate the neuroinflammatory response via the communication between CNS and gastrointestinal immune cells as well as cytokines (105).

DAMPs take the lead to activate the innate immune response in the early stage of IS, followed by the release of brain-derived antigens, leading to adaptive immunity. Immune cells such as neutrophils, monocytes/macrophages (MMs), natural killer (NK) cells, and lymphocytes are recruited from the gastrointestinal tract to the ischemic brain tissues after IS, where they become activated and participate in post-stroke inflammation. Neutrophils, the earliest responders, enter the brain rapidly within hours post-stroke. In the AIS patients, neutrophils were recruited to ischemic regions within twenty-four hours (106). Neutrophil infiltration into the ischemic hemisphere is followed by the release of MMP, NO, and ROS, which exacerbates disruption of BBB and leads to secondary brain injury, resulting in more immune cell infiltration (106–108). Neutrophils’ depletion with antibodies reduces the cerebral infarct volume, alleviates brain edema, and improves neurological function in the experimental IS models (109, 110). MMs migrate and infiltrate the brain via the CCL2-CCR2 axis, which also promotes secondary brain injury and neuroinflammation after IS (111). MMs in the ischemic brain tissues increase from the first day. Then they reach peaks on the third day (112). In mice, the Ly6C^high^ monocyte subset is recruited into the ischemic brain by CCL2 and becomes the typically activated M1 macrophages. In contrast, the Ly6C^low^ monocyte subset expresses CX3CR1 and lacks CCR2 expression. This anti-inflammatory Ly6C^low^ monocyte subset is recruited into the ischemic brain by CX3CL1 and develops into M2 macrophages (113, 114). However, the mechanisms that regulate subsets and the timing of migrating into the ischemic brain are currently unknown. Furthermore, administration of clodronate liposomes decreases the migration of MMs into the brain, reduces the cerebral infarct volume, and promotes neurological function recovery (115). NK cells with cytotoxic functions enter the ischemic brain tissues from the GT in response to chemokines released by necrotic neurons and subsequently cause brain damage via cytotoxic effects and secreting several pro-inflammatory cytokines, such as IFN-γ and IL-17 (17, 116, 117). CD8^+^ T cells, cytotoxic cells which mediate many cytotoxic responses, are found in the ischemic brain tissues within three hours post-stroke (118). Subsequently, antigen-specific cells, such as CD4^+^ T cells and B cells, participate in the pathophysiology after stroke much later after activation by brain-derived antigens and antigen-presenting cells (APCs) (14, 119). Many studies show that T cells play a deleterious role in secondary brain damage after stroke (120–122). However, there are also other studies showing that Th2 cells exert neuroprotective effects by secreting IL-4 or brain-derived neurotrophic molecules to promote stroke recovery (123, 124). So, there is the potential to move stroke towards the good direction by switching the Th1 cell phenotype to the Th2 cell phenotype. Additionally, recent studies have shown that regulatory T cells (Tregs), a class of anti-inflammatory immune cells, are elevated following IS. Tregs account for more than 30% of colonic lamina propria CD4 T^+^ cells and 20% of small gastrointestinal CD4 T^+^ cells (125). Tregs have neuroprotective effects and improve the prognosis of IS by producing anti-inflammatory factors (126–128). For example, Tregs maintain the integrity of gastrointestinal barrier by promoting intestinal stem cells differentiation into intestinal epithelial components via secreting IL-10. On the contrary, Tregs deficiency often leads to a decreased proportion of differentiated intestinal epithelial cells (129).Next, we would like to discuss the role of one type of innate lymphocyte mainly distributed in the GT, γ-δ T cells, in stroke in detail.

γ-δ T cells make up 30% of all T cells in the GT (130). Following IS, γ-δ T cells infiltrate ischemic brain tissues and meninges and are involved in the neuroinflammation and immune responses to cerebral ischemic injury (5, 131, 132). Notably, in one study, γ-δ T cells only accumulated in the meninges, but not in regions of ischemic brain tissue, after stroke (131). However, two other studies suggested that γ-δ T cells infiltrated ischemic brain tissues. The factors responsible for this variability remain unclear (133, 134). In the experimental IS models, γ-δ T cells exacerbate the pro-inflammatory responses and contribute to poor prognosis through IL-17 production (133). Additionally, the migration of γ-δ T cells is dependent on the presence of CCR6. Genetic defects in CCR6 result in significantly reduced migration of γ-δ T cells to the ischemic brain and leptomeninges, along with improved neurological outcomes (134). Nonetheless, the precise mechanisms underlying γ-δ T cell migration require further investigation. Furthermore, γ-δ T cells exert critical effects on the brain tissue injury after IS not by themselves, but by producing all kinds of cytokines, with IL-17 being the most notable.

Small amounts of IL-17 were produced by Th17 cells, but large amounts of IL-17 are generated during acute infection by γ-δ T cells, which rapidly induces inflammation (135). IL-17 is a cytokine family that consists of six members, from IL-17A to IL-17F. IL-17A, one of the members of the IL-17 cytokine family, has been extensively studied in recent years. IL-17A starts to increase from the first day, reaches its peak on the third day, and then then gradually decrease in the experimental IS models (136). IL-17A brain endothelial cells and glial cells to secret a large amount of secretion of CXCL1 and CCL2 by binding to IL-17R up-regulated after experimental IS, thereby promoting the recruitment of immune cells (137). In addition, IL-17A aggravates neuronal ischemic brain injury by mediating excessive autophagy via the Src-PP2B-mTOR pathway (138). These findings show that IL-17A plays a pro-inflammatory effect. Interestingly, IL-17A increases again and reaches a second peak at 28 days post-stroke, mainly produced by astrocytes, and exerts beneficial effects. The effects of IL-17A may be that maintain the survival of neural precursor cells, and enhance neuronal differentiation and synapse formation, which are beneficial for functional recovery after IS (139). Consequently, further studies are required to elucidate the effects of IL-17A in IS patients and the underlying mechanisms that could be targeted for effective clinical therapies. However, it is important to note that most of the supporting evidence originates from experimental studies, and there are discrepancies and unconfirmed phenomena in human stroke. Thus, future research should focus on better clinical translation and the application of experimental findings in clinical practice.

### 5.2 PAMPs induced bacterial inflammation and ischemic stroke

Besides abundant immune cells, GT harbors also a complex community of commensal microbiota (89). Severe ischemic stroke disrupts the balance of the GT microbiota, leading to gastrointestinal dysbiosis. This dysbiosis triggers bacterial inflammation and immune responses in the gastrointestinal tract due to the translocation of microbiota and PAMPs into the bloodstream (11). The term “bacterial inflammation” refers to the immune system’s activation in response to the presence of gastrointestinal microbiota and their metabolites in the bloodstream, which occurs as a result of increased intestinal permeability (140). Then dysbiosis-induced inflammation and immune activation can lead to the release of pro-inflammatory cytokines and oxidative stress, further exacerbating brain injury (11). Additionally, the bacterial inflammation disrupts the gastrointestinal immune homeostasis and further leads to gastrointestinal dysbiosis and more ectopic bacteria. Consequently, ectopic gastrointestinal bacteria and many immune cells enter ischemic brain tissues through the damaged BBB, exacerbating ischemic brain injury (141). Moreover, IS-induced gastrointestinal dysbiosis increase the infiltration of γ-δ T cells from GT into the meninges and ischemic brain (131, 142). The accumulation of γ-δ T cells leads to secret more IL-17, further inducing ischemic brain tissues injury (142). Animal experiments involving the transplantation of fecal matter from ischemic stroke mice into sterile mice have shown that the transplanted feces lead to greater immune cell infiltration, increased cytokine expression in the ischemic brain tissues, and larger cerebral infarction size compared to sterile mice not receiving fecal transplantation (5, 143). This suggests that immune system activation triggered by post-stroke microbiota translocation may result in excessive immune cell activation and subsequent migration to ischemic brain tissue. In addition, gastrointestinal bacteria translocate into the blood circulation following disruption of the gastrointestinal barrier. Subsequently, intestinal bacteria will invade normal sterile tissues and internal organs along with the blood stream, potentially causing gut-derived sepsis and infections at various sites (24, 95). Severe infections occurring either early or late after ischemic stroke are associated with an increased risk of death and poorer prognosis, independent of treatment and baseline characteristics (144). Therefore, it is crucial to actively repair the damaged gastrointestinal barrier after stroke to prevent further deterioration of the gastrointestinal microenvironment, halt the mutually reinforcing processes, decrease the incidence of post-stroke infections, and improve stroke prognosis. Meanwhile, close monitoring the blood composition in stroke patient is also important to prevent post-stroke infectious complications. For example, in a clinical study, researchers found that the red cell distribution width-albumin (RA) ratio predicted stroke-related infections and mortality in stroke patients (145). Moreover, although prophylactic antibiotics don’t improve functional outcomes versus mortality, they significantly reduce the overall risk of infections, particularly urinary tract infections (94, 146).

### 5.3 The impact of GBMA on the development of stroke risk factors

The development of risk factors for ischemic stroke (IS) is influenced by various complex factors, including the gut microbiota (GM). In particular, GM has been found to impact several key risk factors associated with IS.

GM and hypertension: Hypertension, a common risk factor of IS, has a critical induction effect on IS and is closely related to the prognosis of IS (147, 148). Studies have revealed that GM metabolites regulate blood pressure (BP) levels. SCFAs modulate vasodilation of intestinal vessels to affect BP by binding with GPR41 (57, 149). Conversely, hypertension affects the composition of GM, leading to significant alterations and imbalances. The abundance of gastrointestinal microbiota decreased significantly and was unbalanced in the animals and humans with hypertension (149, 150). Additionally, hypertension is associated with decreased levels of tight junction proteins in the gastrointestinal tract, resulting in the increased permeability of the gastrointestinal barrier (151). Trimethylamine oxide (TMAO), a metabolite of gut microbiota, affects BP by prolonging the duration of angiotensin II action and increasing water reabsorption via the TMAO-AVP-AQP-2 signaling pathway (152, 153). Increased TMAO levels in the patient’s circulation are closely correlated with the elevated levels of CD14 ^+^ and CD16 ^+^ pro-inflammatory monocyte, which may contribute to an increased risk of cardiovascular events (154).

GM and hyperlipidemia: Hyperlipidemia are associated with increased occurrence of cardiovascular events and poor stroke prognosis. The interplay between GM and cholesterol levels has been observed, with specific GM taxa such as Erysipelotrichaceae, Bacteroides, Alistipes, and Pasteurella positively correlating with plasma cholesterol levels (155). Hyperlipidemia also influences peripheral inflammatory and immune responses. Hyperlipidemia increases the generation of hematopoietic progenitors from the bone marrow, leading to an increase in the population of MMs and neutrophils in the peripheral circulation (156). Ly6C^high^ proinflammatory monocytes are observed in hyperlipidemic mice (157). Moreover, hyperlipidemia regulates lymphocyte functions. For example, LDL uptake by APCs stimulates naive T cells to differentiate into Th1 cells while reducing the activity of anti-inflammatory Th2 cells (158). In addition to affecting inflammation and immune responses, hyperlipidemia affects tissue repair and remodeling processes by influencing the clearance of pathogens and injured tissues (158).

GM and atherosclerosis: Recent studies have shown that elevated levels of TMAO have been associated with the probability and severity of cardiovascular events (159, 160). Moreover, the level of TMAO is also related to the size of atherosclerotic plaque (161). This shows that microorganisms, TMAO and atherosclerosis have a certain relationship, but at present, little is known about the specific molecular mechanism of how TMAO mediates atherosclerosis. Some studies have found that DNA of bacteria is found in atherosclerotic plaque. The bacteria also exist in gastrointestinal microorganisms, suggesting a potential role of gut microorganisms in the development of atherosclerosis (162). Recent clinical trials have demonstrated significant changes in the composition of GM after stroke, particularly an increase in the proportion of harmful opportunistic pathogens in individuals with atherosclerosis (9).

GM and diabetes: Patients with diabetes have a high incidence of stroke and a generally poor prognosis (163). Altered GM composition contributes to changes in insulin levels and insulin resistance by interfering with the insulin signaling pathway (164). Animal experimental studies suggested that acetate might reduce the frequency of autoreactive T cells in lymphoid tissues of diabetic mice. In contrast, butyrate increases the number and promotes the function of Tregs by inhibiting HDAC9 and increasing the key gene transcription of Tregs (165). Dietary interventions involving butyrate and acetate have shown beneficial effects on GM integrity, Treg numbers, and cytokine levels (165). Moreover, diabetes disrupts the balance of T cell differentiation, leading to an increased proportion of pro-inflammatory Th1 and Th17 cells, while reducing the proportion of anti-inflammatory Th2 cells and Tregs (166, 167).

GM and aging: Aging is an inevitable risk factor for stroke, and elderly individuals have a higher mortality rate following stroke, regardless of the extent of brain damage (168). Compared with healthy young adults, the gastrointestinal microbiota of the elderly is significantly changed (169). The gastrointestinal microbiota of the elderly significantly differs from that of healthy young adults, with a decline in diversity, decreased abundance of commensal bacteria, and an increase in potentially harmful pathogens. These changes may contribute to gastrointestinal inflammation and disruption of immune homeostasis (170, 171). Moreover, recent study has provided evidence indicating that in elderly individuals, the deterioration of intestinal barrier function contributes to the increased translocation of bacteria from the gut, thereby heightening the risk of bacterial infection following stroke (172). Additionally, aging downregulates innate and adaptive immunity upon CNS injury. Aging reduces phagocytic activities of MMs, chemotaxis of neutrophils, and cytotoxicity of NK cells (23, 173–175). T and B lymphocytes also undergo alterations in the number and function, and the differentiation of memory B cells into plasma cells is impaired in older individuals (176, 177).

GM and micronutrient deficiencies: Micronutrient supplementation for stroke prevention has been extensively studied, but the results have been inconclusive in terms of safety and efficacy (178–181). Some studies have suggested a potential reduction in the risk of ischemic stroke with certain vitamin supplementation. However, the underlying mechanisms by which vitamins exert their protective effects against stroke risk remain uncertain (182).

In conclusion, the interplay between GBMA and these risk factors has a significant impact on the occurrence and development of stroke. Therefore, active secondary prevention strategies should be implemented following stroke to halt further deterioration, improve prognosis, and reduce the likelihood of recurrence. It is equally important to consider the influence of GBMA on stroke risk factors, not only focusing on post-stroke changes in the gastrointestinal microenvironment, but also monitoring and managing alterations in gastrointestinal microbiota and function before a stroke occurs. Further exploration of pre-stroke gastrointestinal management and interventions is necessary to better understand and proactively address changes in the gastrointestinal microenvironment prior to stroke onset.

## 6 Clinical evidence of brain and GT interactions after stroke

In recent years, the role of the brain gut axis after stroke has become a research hotspot, therefore, clinical evidence of the crosstalk between the post-stroke brain and the GM and gastrointestinal immune system has gradually increased (**Table 2**). After stroke, the probability of intestinal dysfunction in patients increased sevenfold (186). Some fecal samples from acute ischemic stroke patients all suggest that opportunistic pathogens from the patient’s gut show a variable degree of enrichment and decrease in the abundance of SCFA producing bacteria (8, 9, 93, 185). These alterations appear to increase the risk of adverse functional outcomes.

**Table 2:** Summary of clinical evidence about brain and GT interactions after stroke.

Patients	Controls	Samples	Finding	Reference
297 acute ischemic stroke patients and 25 transient ischemic attack (TIA) patients	3 subgroups: AS: 48 participants with obvious carotid plaques; IMT: 104 participants with increased intima–media thickness of the carotid arterial wall; Non-AS: 79 participants with healthy carotid arteries.	Fasting blood samples: Patients: 322 blood samples; Controls: 231 blood samples. Fecal samples: Patients: 141 fecal samples within 48 hours after admission; Controls: 97 fresh fecal samples.	Compared with asymptomatic atherosclerotic controls, patients with IS and TIA showed significant dysbiosis of the gastrointestinal microbiota and had lower blood levels of TMAO.	(183)
41 ischemic stroke patients	40 control subjects	81 blood samples and 81 fecal samples.	Gut dysbiosis in patients with IS is associated with host metabolism and stroke-induced inflammation.	(91)
20 patients with AIS and 10 patients with 15 days of treatment	16 healthy volunteers	46 fresh fecal samples of all participant.	Gut microbiota status differs between stroke patients or healthy individuals in different states.	(184)
140 acute ischemic stroke patients	92 healthy controls	Patients: 140 fresh fecal samples of patients within 72 hours after and blood samples; Controls: fresh fecal samples and blood samples.	AIS patients develop gut dysbiosis, and dysbiosis of gut microbiota that produce SCFAs, which increases the risk of poor functional outcomes.	(8)
132 acute ischemic stroke patients	No	132 fecal and fasting blood samples taken within 24 hours after admission	Poor functional outcome at 3 months of the acute phase of stroke was associated with increased pathogenic bacteria, decreased SCFAs producing bacteria, and up regulation of membrane transport and xenobiotic biodegradation pathways.	(185)
349 ischemic (287) and hemorrhagic (37) stroke patients	51 healthy controls	Patients:349 fecal samples taken within 24 hours after admission and 35 fasting blood samples; Controls: 51 fecal and fasting blood samples	The degree of gut microenvironment disruption is directly proportional to stroke severity. Lower abundance of butyrate producing bacteria within 24 hours of admission was an independent predictor of increased risk of post-stroke infection.	(93)
36 stroke patients	10 healthy controls	Fasting blood samples	>70% of the microorganisms detected in the infected stroke patients were commonly known to be commensal bacteria that reside in the human intestinal tracts	(18)
30 cryptogenic stroke patients	33 healthy controls	Patients:30 stool samples taken within 48 hours after admission and 26 intestinal biopsy specimens; Controls: 33 fecal and fasting blood samples and 25 intestinal biopsy specimens	Gut dysbiosis in CS patients was associated with the severity of CS and the systemic inflammation. Maintaining the intestinal homeostasis may be a potential strategy for the treatment of CS.	(10)
98 ischemic stroke patients	No	No	Bowel dysfunctions increases significantly after stroke	(186)
28 patients with acute ischemic stroke	28 healthy controls	Fecal samples at 0–4, 5–7, and 8–30 days, and 1–4 months post stroke.	IS rapidly triggers gut microbiome dysbiosis with Enterobacteriaceae overgrowth that in turn exacerbates brain infarction.	(11)

In a clinical study, it was observed that stroke patients generally exhibit increased levels of plasma c-reactive protein (CRP), lipopolysaccharide (LPS), LPS-binding protein, and white blood cell counts. These findings suggest the presence of a systemic inflammatory response in stroke patients. Additionally, the prevalence of gastrointestinal disorders, gastrointestinal inflammation, and gastrointestinal permeability was found to be higher in a group of 26 stroke patients compared to the control group of 25 healthy individuals. These results indicate a close association between gastrointestinal disorder in stroke patients, stroke severity, and systemic inflammation. However, it is important to note that this clinical study had limitations due to the small number of subjects included (10).

Another clinical study indicates that a higher abundance of opportunistic pathogens and a lower abundance of butyrate producing bacteria are observed in the GM of individuals at high risk of stroke relative to low-risk healthy individuals (187). In another clinical study, researchers separately collected data on the GM of 16 healthy people, 20 patients with stroke, and 10 patients treated for 15 days after acute cerebral infarction, hoping to reach the goal of diagnosing ischemic stroke by identifying the characteristics of the microbiota (184). When considering these two clinical studies together, it suggests that the microbiota of individuals at high risk of stroke may be undergoing insidious changes slowly compared to healthy individuals. The onset of stroke acts as a triggering point that rapidly amplifies this gradual process, leading to a significant alteration in the gastrointestinal microenvironment. This amplification process occurs through a positive feedback mechanism of crosstalk between the brain and the GT, wherein the three components mutually reinforce each other, thereby exacerbating the prognosis of stroke. Of course, this at the same time provides novel therapeutic strategies based GBMA, where we can stop the aggravation of IS by interrupting the positive feedback effect among three components.

## 7 GBMA based therapeutic strategies

### 7.1 Targeting receptors and blocking the nerve conduction

After IS, communication between CNS and the GT occurs through various pathways, including SNS, PNS, ENS, and HPA axis. Certain drugs can be used to modulate this crosstalk by targeting proteins and receptors involved in the brain-gut communication and inhibiting nerve conduction.

Following IS, there is an imbalance between gastrointestinal adrenergic signaling and cholinergic signaling, with a shift towards increased adrenergic signaling and reduced cholinergic signaling (18). Adrenergic signaling has been associated with systemic immunosuppression after stroke, which increases the risk of post-stroke infections (188, 189). On the other hand, reduced cholinergic signaling promotes proinflammatory immune responses (189, 190). Therefore, the application of drugs blocking adrenergic receptors or stimulating cholinergic receptors may be a viable approach to prevent the formation of positive feedback. For example, the use of β-adrenergic receptor blockers has been shown to significantly decrease bacterial presence in the lungs, liver, and spleen of stroke mice (18). However, the effects of post-stroke β-adrenergic receptor blocker therapy on stroke treatment outcomes have shown variability (**Table 3**). Therefore, in the clinical application of β-blockers for stroke treatment, careful consideration of the timing of administration is necessary to counteract the systemic immunosuppressive effects induced by SNS activation and enhance the immune response to prevent post-stroke infections.

**Table 3:** Some relevant researches on the application of receptor blockers after stroke.

Objects	Drugs	Results	Conclusion	Reference
Mouse (MCAO)	Propranolol or metoprolol	A significant reduction in the bacteria that were detectable in the lung, BALF, liver and spleen of post-stroke mice	Blockade of β-adrenergic receptors reduces gut permeability and post-stroke infections.	(18)
Mouse (MCAO)	Propranolol RU486	Propranolol reduce or prevent bacterial dissemination at 72 h after MCAO. However, delayed administration of propranolol neither increased IFN-γ production nor decreased bacterial load. RU486 had no effect on blood or pulmonary bacterial burden	Propranolol administered in the early stage of stroke can prevent bacterial infection in mice with MCAO and reduce the mortality of mice. Delayed administration after MCAO cannot prevent bacterial infection.	(47)
Mouse (MCAO)	Propranolol RU486	Treatment with propranolol and RU486 preserved at a greater extent the number of NK cells and their expression of CD69 and IFN-γ and lower LM burden than with drugs alone	Propranolol and RU486 synergistically suppress post-stroke immunosuppression and prevent infection	(191)
Rat (MCAO)	Carvedilol propranolol prazosin	Carvedilol administration reduced infarct volume. Propranolol administration had no effects on stroke outcome Prazosin administration decreased the levels of TNF-α in the spleen but didn’t affect the infarct volume.	Propranolol administration didn’t provide neuroprotection after MCAO.	(192)
Mouse (MCAO)	Propranolol	Propranolol administration reduced poststroke infection.	Propranolol administration reduced post-stroke infection.	(188)
Human (553 IS patients and 72 hemorrhagic stroke patients)	β-adrenergic receptor blockers	Treatment with β-adrenergic receptor blockers was not associated with a lower risk for post-stroke pneumonia. However, there is a significant risk reduction of 35% for urinary tract infections in patients treated withβ-adrenergic receptor blockers.	β-blocker therapy did not reduce the risk for post-stroke pneumonia, but significantly reduced the risk for urinary tract infections.	(193)
Human (5212 IS patients)	β-blocker (BB) therapy	On-stroke BB was associated with reduced mortality. Both pre-stroke and on-stroke BB were associated with reduced frequency of pneumonia.	On-stroke BB was associated with reduced mortality. Pre-stroke and on-stroke BB were inversely associated with incidence of nosocomial pneumonia.	(194)
Human (1375 IS patients)	β-adrenergic receptor blockers	There was no difference in stroke severity between nonusers and those on either a selective β1 blocker or a non-selective β blocker.	Pre-stroke use β receptor blockers do not appear to affect stroke severity and functional outcome at 3 months	(195)

In the context of traumatic brain injury, enhancement of cholinergic signaling prevents increases in gastrointestinal and cerebrovascular permeability (196, 197). The mechanism has not been demonstrated in the ischemic stroke. However, a study suggests that targeted vagus nerve stimulation can have complex effects in reducing neuroinflammation (198). Moreover, the release of acetylcholine (Ach) is enhanced by electrical stimulation of the VN, which acts on α7 nicotinic acetylcholine receptors of macrophages to inhibit the release of proinflammatory cytokine, such as TNF and attenuate the serum TNF response induce by LPS after stroke-induced GM translocation (199). CNI-1493, an anti-inflammatory agent, causes deactivation of macrophages and inhibits the synthesis of pro-inflammatory mediators. Intracerebroventricular injection of CNI-1493 after IS inhibits TNF synthesis in the brain. However, the inhibitory effect of CNI-1493 on LPS induced TNF synthesis was counteracted when VN conduction was disrupted through surgery or the application of atropine (200, 201). When considering the translation of research findings to clinical applications, the process of using drugs to interrupt positive feedback might be feasible in the course of future clinical studies. However, it is crucial to focus on the development of more precise receptor blockers or agonists and to carefully consider the timing of drug administration. Additionally, completely cutting off nerve conduction is not a feasible approach, but these findings have important implications for the potential treatment of stroke and warrant further investigation in clinical settings.

### 7.2 Targeting immune cells and cytokines

The gastrointestinal microbiota, immune cells, and cytokines are interconnected components within the complex network of post-stroke inflammation. Dysbiosis of the gastrointestinal microbiota following stroke can initiate immune cell activation and subsequent release of pro-inflammatory cytokines, exacerbating neuroinflammation. Numerous animal experiments have highlighted the potential therapeutic strategy of targeting immune cells and cytokines to modulate the inflammatory response and mitigate neuroinflammation in stroke. Antibody-mediated neutrophil depletion has shown promising effects in experimental ischemic stroke, including reductions in cerebral infarct volume, alleviation of brain edema, and improvement in neurological function (109, 110). Likewise, the use of clodronate liposomes, which effectively reduce monocyte-derived macrophages (MMs) by 80%, has resulted in decreased migration of MMs into the brain, decreased cerebral infarct volume, and facilitated recovery of neurological function (115). Blockade of T cell infiltration through the use of immunosuppressants such as FTY720 or the targeted elimination of γ-δ T cells and IL-17 T lymphocytes in the brain using TCRγδ during the subacute stage of cerebral infarction has shown improved outcomes in ischemia-reperfusion injury (133). However, it is important to consider the potential impact of targeting immune cells on the overall immune function of the post-stroke organism, as this can affect the occurrence and development of post-stroke infection. For example, reducing blood neutrophils may increase the risk of post-stroke infection due to the presence of post-stroke systemic inflammatory response syndrome (SIS). Clinical studies have found that impaired neutrophil function is associated with the development of post-stroke infection, but further research is needed to determine whether neutropenia influences the development of post-stroke infection in the context of post-stroke immunosuppression (202). Therefore, additional studies are required to investigate the feasibility of targeting immune cells for post-stroke therapy and to support risk assessment of post-stroke infection in clinical practice.

Following immune system activation triggered by stroke, certain immune cells play a neuroprotective role. Regulatory T cells (Tregs) contribute to neuroprotection by maintaining immune homeostasis and balancing the release of anti-inflammatory and pro-inflammatory factors. Modulating the number and functions of Tregs has been identified as a potential therapeutic approach. For instance, pretreatment with resveratrol has been shown to increase Treg production and accumulation before stroke onset, offering partial protection to ischemic brain tissues (203). Furthermore, modulating the gastrointestinal microbiota presents a promising approach to influence immune cell activation after stroke. Achieving gastrointestinal microecological homeostasis through fecal microbiota transplantation (FMT) increases the number of Tregs, which primes a protective immune response after stroke (143). Another group of immune cells, known as regulatory B cells (Bregs), also exhibit neuroprotective effects by suppressing inflammatory responses and modulating immunity. Pretreatment with lipopolysaccharide (LPS) increases Bregs populations in post-stroke mice, leading to the attenuation of inflammatory response and neurological damage. Specifically, the expansion of marginal zone B cells and follicular B cells in mice is associated with these neuroprotective effects (204). In addition to the aforementioned experiments, various other immune cells and cytokines can serve as potential therapeutic targets, as detailed in **Table 4**. While the precise mechanisms and optimal therapeutic targets are still being elucidated, targeting immune cells and cytokines holds promise as a therapeutic approach for attenuating neuroinflammation and promoting post-stroke recovery. It is important to note that there is still a considerable gap between experimental research and clinical translation, requiring further investigation and validation.

**Table 4:** Treatment of ischemic stroke with immune cells or cytokines as targets.

Cells/cytokines	Treatment	Effects	Reference
T cells/IFN-γ/IL-10/IL-4	IL-33	Reduce the production of IFN-γ. Increase Foxp3+ T cells and the secretion of IL-4, IL-10 and the transforming growth factorβ (TGF-β).	(124, 205)
Neutrophils	Anti-neutrophil antibody (Ab) and G-CSF	Reduce the number of neutrophils. Prevented brain atrophy and significantly improve neurological function.	(109)
Macrophages	Clodronate liposomes	Deplete macrophages and reduce macrophage infiltration into brain tissues. Promote post-stroke tissue repair and remodeling.	(115)
MMs/TNFα/IL-6/IL-1β/MCP-1	αCD147	Inhibit stroke-induced inflammatory response by decreasing the number of Ly-6Chigh MMs subset and reducing expression of TNFα, IL-6, IL-1β and MCP-1.	(206)
TNF-α/microglial	Etanercept	Inhibit TNF-α as well as downregulate the activation of brain microglial.	(207)
IL-6	Tocilizumab	Inhibit IL-6 and reduce neuronal cell apoptosis.	(208)
IL-17A	Monoclonal antibody against IL-17A	Inhibit astrocyte activation in the infarct area of mice and alleviate of ischemic brain injury.	(209)
Tregs	IL-2 and IL-2 antibody complex (IL-2/IL-2Ab)	Increase Tregs in the peripheral circulations and enhance their neuroprotective effects by increasing the expression of CD39 and CD73 in the Tregs.	(210)

### 7.3 Fecal microbiota transplantation

After IS, gastrointestinal dysbiosis occurs, leading to the translocation and alteration of the gastrointestinal microbiota, which can significantly influence the severity of brain damage. Therefore, correcting dysbiosis in acute ischemic stroke (AIS) patients through fecal microbiota transplantation (FMT) appears to be a feasible strategy to restore gastrointestinal (GT) homeostasis (211, 212). Notably, FMT enriched with SCFAs has shown positive effects on cerebral ischemia in experimental IS models by remodeling the gastrointestinal microbiota, enhancing the abundance of beneficial Lactobacillus species, and improving the gastrointestinal microenvironment (213). In severe stroke patients, the gastrointestinal microbiota undergoes significant changes following stroke, contributing to immune homeostasis disturbances. FMT has demonstrated considerable improvements in outcomes and survival rates among these severe IS patient (5). It is important to acknowledge that, like any medical intervention, FMT carries potential adverse effects such as nausea, vomiting, abdominal discomfort, low-grade fever, flatus, and alterations in bowel habits, which are generally self-limiting in nature (214). The underlying mechanisms through which FMT improves the outcome of IS remain largely unknown, although it is generally believed that restoration of intestinal homeostasis plays a pivotal role. Further research is necessary to elucidate the specific mechanisms involved. Additionally, individualized characteristics of the gastrointestinal microbiota should be taken into consideration. It is plausible that there may be differential alterations in the gastrointestinal microbiota among individuals after stroke, necessitating tailored treatment regimens based on individual differences at the time of intervention. Furthermore, given that the gastrointestinal microbiota may also contribute to disease, careful selection of appropriate healthy FMT donors is of utmost importance.

### 7.4 Probiotics and prebiotics treatment

Probiotics are live microorganisms that, when administered in adequate doses, confer health benefits on the host. Prebiotics’ definition is selectively used substrates by host microorganisms, which are beneficial to the host’s health (215). In Section 2.4, we discussed the crucial roles of short-chain fatty acids (SCFAs) and acyl-homoserine lactones (AHLs) in the gut-brain-microbiota axis (GBMA). Numerous animal experiments and clinical studies have demonstrated that probiotic and prebiotic treatments can enhance the production of SCFAs and AHLs, indirectly influencing the prognosis of stroke. Probiotics derived from food sources, when introduced into the gastrointestinal tract (GT), increase the abundance of SCFA-producing microorganisms and promote SCFA production (216). Furthermore, probiotics have been shown to improve post-stroke gastrointestinal complications such as constipation, abdominal distension, and diarrhea (217). Daily oral administration of probiotics also rebalances the Th1/Th2 immune response and improve neuronal function in patients with severe traumatic brain injury (218). Prebiotics, specifically carbohydrates, can influence the production of SCFAs and mucin, as well as modulate the local inflammatory response of the gut-associated lymphoid tissue (GALT) (219). High amylose maize starches are used as a substrate for bacterial fermentation, which increases the production of SCFAs and fecal concentrations of acetate and butyrate (220). Supplementation with lactulose, a common prebiotic, has been shown to repair gut barrier damage, mitigate gut microbiota dysregulation, and partially improve functional outcomes after stroke e (221). Additionally, a clinical study demonstrated that the use of functional barley as a prebiotic increased the abundance of butyric acid-producing bacteria, consequently elevating butyric acid levels in the GT (222). Moreover, the combination of probiotics and prebiotics can harness the synergistic effect between the two. In an animal study, co-administration of inulin and SCFA-producing bacteria increased SCFA production and improved neurological deficit scores and behavioral outcomes in post-stroke mice compared to the administration of SCFA-producing bacteria alone (6). Another animal experiment showed that the combination of Li01 and resveratrol improved microbiota composition and metabolism, increased SCFA and AHL production, and enhanced the prognosis of colitis (223). Based on these findings, it can be hypothesized that probiotics and prebiotics may improve the prognosis of ischemic stroke patients by influencing the production of SCFAs and AHLs. In summary, probiotics and prebiotics have the potential to improve the composition of the microbiota and gastrointestinal function, thereby positively impacting the prognosis of stroke patients. While probiotic and prebiotic therapies are already being used in clinical practice for stroke treatment and secondary prevention, prospective clinical intervention trials are still needed to establish their effectiveness.

### 7.5 Diet therapy

Diet plays an essential role in influencing the composition of gastrointestinal microbiota. Consuming high amounts of phosphatidylcholine and L-carnitine in the diet increases the production of trimethylamine N-oxide (TMAO), potentially raising the risk of cardiovascular event (224). Antibiotic treatment has been shown to reduce TMAO production by altering the composition of the gut microbiota, while controls without antibiotics exhibited elevated TMAO levels (154). The specific mechanisms through which ketogenic diets modulate the gastrointestinal microbiota to improve outcomes in ischemic stroke (IS) are not yet fully understood. However, several studies provide valuable insights for further exploration of the relationship between ketogenic diets, microbiota modulation, and stroke outcomes. Ketogenic diets significantly increase GABA levels in the hippocampus, which is related to the regulatory effects of gastrointestinal microbiota (225). Early intervention with ketogenic diets improves the gastrointestinal microbiota, enhances cerebrovascular function, improves metabolism, and promotes the elimination of β-amyloid, thus reducing the risk for Alzheimer’s disease (226).

There is a growing interest in understanding the impact of dietary fiber on the composition of the GM. Dietary fiber is primarily digested by Firmicutes upon entering the gastrointestinal tract, leading to increased production of SCFA (227). Animal experiments have demonstrated that adding butyric acid to the diet is effective for treating IS (228). Moreover, clinical studies have shown that short-term vegetarian diets increase the abundance of Roseburia, Ruminococcus, and butyrate-producing bacteria in the gut microbiota (229). The impact of SCFAs on post-stroke development and recovery is reviewed in detail above. Therefore, supplementing SCFAs and precursors of SCFAs in the diet may be an excellent therapeutic strategy (51). Taken together, diet therapy improves microbiota composition and intestinal function and then the prognosis of stroke patients.

## 8 Conclusion

The advancing research in the field has gradually unveiled the intricate crosstalk between the brain and gastrointestinal tract (GT). As previously discussed, the involvement of the gut-brain-microbiota axis (GBMA) in stroke onset and progression emphasizes the interconnectedness of these systems following a stroke. In this section, we delve deeper into the mutual crosstalk among the three components of GBMA and explore how the formation of a vicious circle contributes to a poor prognosis in stroke. However, while our understanding of the role of GBMA in stroke has expanded, the precise cellular, signaling, and molecular mechanisms underlying its influence remain to be fully elucidated. Further research is required to unravel these intricate mechanisms.

One of the unresolved aspects is the differential effects exerted by GBMA at different stages of ischemic stroke, particularly when various immune cells infiltrate the ischemic brain tissue. The specific molecular mechanisms that govern these differential effects are not yet clear. Consequently, there is an urgent need to intensify preclinical studies focusing on developing new therapies for the prevention and treatment of ischemic stroke. Additionally, the combination of potential therapeutic strategies with rtPA thrombolytic therapy has been proposed as a means to minimize post-stroke injury. However, translating the findings from animal experiments and clinical studies into clinical practice poses several challenges.

Although numerous reports have shed light on the alterations in gastrointestinal microbiota after stroke and the bidirectional communication between GBMA components, many specific molecular mechanisms remain poorly understood. For instance, while stroke leads to increased gastrointestinal permeability, it is not yet elucidated whether stroke alters the expression of critical tight junction proteins associated with gastrointestinal permeability. Furthermore, although the impact of stroke on the gastrointestinal microbiota is well-documented, the precise mechanisms underlying these alterations are still largely unexplained. We have yet to determine the molecular mechanisms through which certain bacterial metabolites affect the development and prognosis of stroke. Addressing these gaps in knowledge will require further investigations at the molecular level.

Moreover, the ultimate goal of future research is to translate the findings from basic experiments into clinical practice. The conclusions drawn from these experiments need to be applied in a clinical setting to have practical significance. However, there are numerous challenges associated with this process of clinical translation. These challenges stem from various factors, such as the complexity of the human body, the heterogeneity of stroke patients, and the limitations in conducting large-scale clinical trials. Overcoming these hurdles is crucial for successfully integrating the knowledge gained from basic research into clinical applications

In this review, we have summarized potential therapeutic strategies based on the exacerbation cycle of GBMA. However, it is important to note that translating the results of animal experiments and clinical studies into clinical practice presents its own set of difficulties. These therapeutic strategies require further validation through long-term clinical studies to determine their feasibility and efficacy. The process of discovering these intricate details and effectively translating them into clinical practice is expected to be an arduous and time-consuming endeavor.

In conclusion, the investigation of the crosstalk between the brain and gastrointestinal microbiota following stroke has provided valuable insights into the pathophysiology of stroke and potential therapeutic approaches. However, there are still many unanswered questions regarding the specific molecular mechanisms and clinical implications of GBMA in stroke. Future research efforts should focus on elucidating these mechanisms and overcoming the challenges associated with clinical translation. By doing so, we can pave the way for the development of novel therapies and interventions that have a meaningful impact on stroke prevention, treatment, and patient outcomes.

## Author contributions

Y-HW and R-TB wrote this manuscript. BH and Y-NL designed the review and edited the manuscript. C-LZ, Y-MQ, and J-ZL provided some suggestions. All authors contributed to the article and approved the submitted version.
